# Platelet-rich plasma -mediated dual repair of immunity and barrier: an innovative hypothesis for the treatment of allergic rhinitis

**DOI:** 10.3389/fcimb.2026.1749953

**Published:** 2026-03-13

**Authors:** Ju Tian, Hongyuan Zhu, Chunhui Ou, Jieyu Yang, Wenjie Fu, Xin Li, Biao Cheng

**Affiliations:** 1 Department of Plastic Surgery, Zhongshan City People’s Hospital, Zhongshan, Guangdong, China; 2 First Clinical Medical College of Guangdong Medical University, Zhanjiang, Guangdong, China; 3 Shenzhen University Medical School, Shenzhen, Guangdong, China; 4 Department of Otolaryngology, Zhongshan City People’s Hospital, Zhongshan, Guangdong, China; 5 Department of Plastic Surgery, General Hospital of Southern Theater Command, People’s Liberation Army, Guangzhou, Guangdong, China

**Keywords:** allergic rhinitis, barrier repair, growth factors, immune modulation, platelet-rich plasma

## 1 Introduction

Allergic rhinitis (AR) is a common chronic disease affecting hundreds of millions of individuals globally, characterized by paroxysmal sneezing, watery rhinorrhea, nasal pruritus, and nasal congestion. It severely impairs patients’ quality of life and increases the risk of complications such as asthma and nasal polyps (Bernstein et al., 2024). These typical symptoms profoundly compromise patients’ daily functioning. The core pathophysiological mechanism of AR revolves around a vicious cycle involving two interconnected processes: first, Th2 cell-mediated immune dysregulation, which drives persistent inflammation; second, dysfunction of the nasal mucosal epithelial barrier, manifested by disruption of tight junctions, which facilitates increased allergen penetration (Yang et al., 2023; Li et al., 2025). Inflammatory cytokines damage the barrier, while barrier leakage exacerbates immune responses, forming an intractable pathological loop (Izquierdo et al., 2022). Current therapeutic approaches for AR exhibit notable limitations (Pavón-Romero et al., 2022; Layhadi et al., 2024; Zhang et al., 2025). Symptomatic treatments such as intranasal corticosteroids and oral anti-allergic agents either focus on anti-inflammation with limited barrier repair capacity or merely provide transient symptom relief, neither achieving a cure (Zhang et al., 2025). Allergen immunotherapy (AIT), though potentially disease-modifying, is characterized by long treatment courses, potential risks, and limited applicability (Pavón-Romero et al., 2022; Layhadi et al., 2024). A critical unresolved issue is that existing therapies fail to simultaneously target both immune dysregulation and barrier disruption, which are two pivotal pathological mechanisms. While some emerging approaches, such as mesenchymal stem cell (MSC) -based therapies, can contribute to barrier repair, they do not comprehensively address both pathological aspects (Işık et al., 2017; Wang et al., 2023), For example, intraperitoneal MSC administration ameliorates allergic rhinitis in murine models by migrating to nasal and lung tissues and suppressing T helper (Th)2 immune responses (Işık et al., 2017), yet a significant unmet clinical need remains. Consequently, an innovative therapy capable of safely and effectively achieving dual repair of immunity and barrier function to fundamentally disrupt the pathological cycle of AR holds substantial clinical and scientific value.

Platelet-rich plasma (PRP), a platelet concentrate prepared via centrifugation of autologous whole blood (Rath et al., 2025), releases abundant growth factors stored in α-granules (e.g., platelet-derived growth factor [PDGF], Transforming growth factor-β1 [TGF-β1], epidermal growth factor [EGF], vascular endothelial growth factor [VEGF], and insulin-like growth factor [IGF]) upon platelet activation (Rath et al., 2025). These factors act synergistically and have demonstrated efficacy in anti-inflammation, promoting cell proliferation, and enhancing angiogenesis in fields such as orthopedic repair, wound healing, and skin regeneration. Recent studies have revealed that PRP alleviates symptoms of atrophic rhinitis (Friji et al., 2014; Mostafa and Ayad, 2020; Kim et al., 2021; Bondarenko and Bezshapochny, 2023; Asiry et al., 2025; Tulaci et al., 2025) and sinonasal polyps (Samiea et al., 2024) through multiple mechanisms, including promoting barrier repair, enhancing ciliary function, exerting anti-inflammatory effects, regulating collagen metabolism, and improving microcirculation, with favorable safety profiles. However, current evidence remains constrained by methodological variability, necessitating more high-quality studies to support its clinical application. Notably, no published research has explored PRP as a treatment for AR to date. Although MSC therapy has shown therapeutic efficacy in murine models of AR (Işık et al., 2017; Wang et al., 2023), PRP offers distinct and notable advantages. These include its autologous origin—eliminating the risk of immune rejection—and its abundant, naturally derived cocktail of growth factors, which can synergistically target the dual pathological hallmarks of AR: immune dysregulation and nasal epithelial barrier damage. Importantly, PRP-based interventions for AR do not involve MSC-based therapies, underscoring its unique role as a standalone therapeutic strategy. Given PRP’s pleiotropic biological properties, we hypothesize that its unique characteristics align well with the therapeutic demand for “dual repair of immunity and barrier function” in AR. This article aims to systematically propose and validate this innovative hypothesis.

## 2 Hypothesis: PRP-mediated “dual repair of immunity and barrier” mechanism

**Figure 1:**
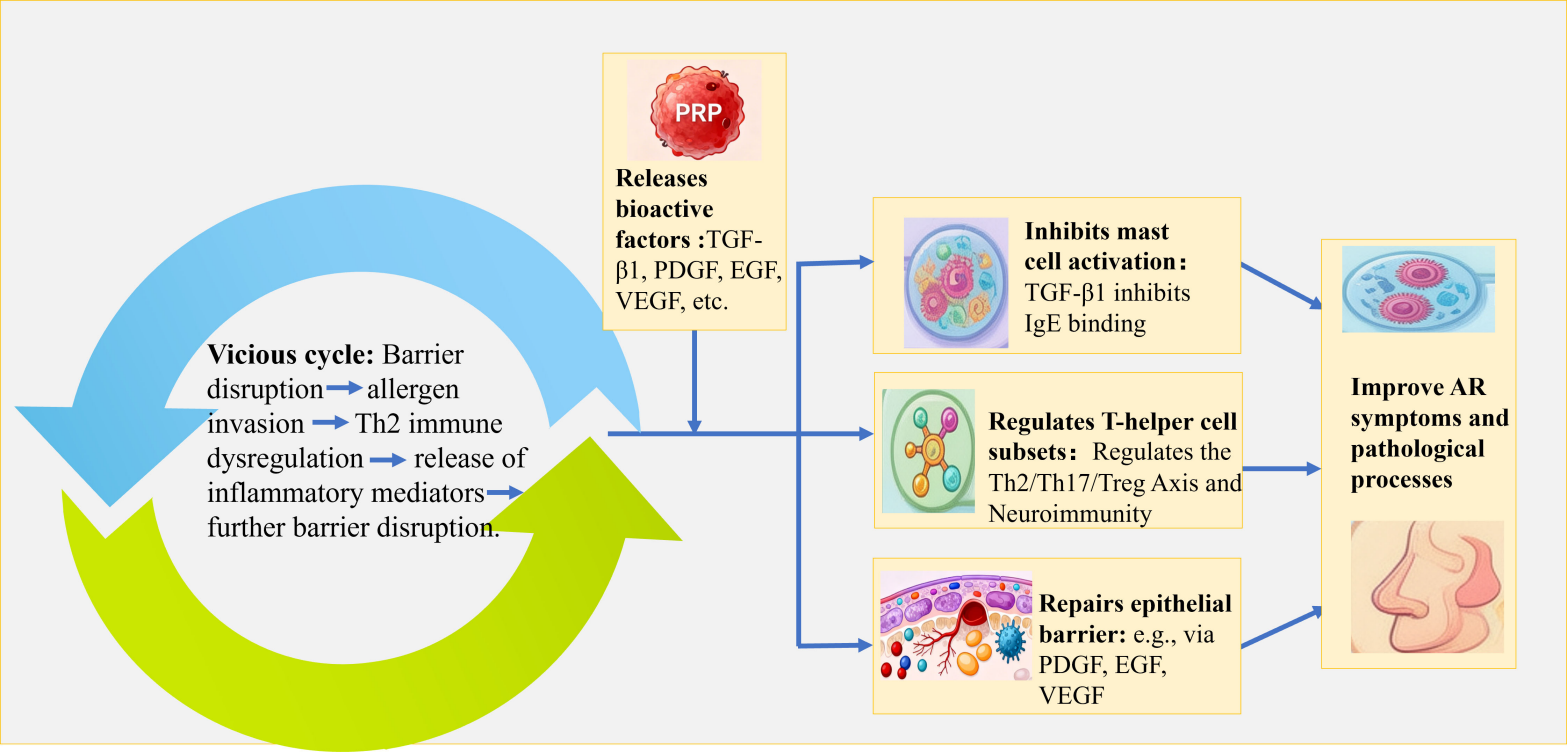
Mechanistic schematic diagram of platelet-rich plasma (PRP)-Mediated “Dual repair of immunity and barrier” for Allergic Rhinitis (AR) treatment.

This hypothesis posits that PRP may intervene in the pathological process of AR by synergistically acting through dual pathways of immune modulation and barrier repair, thereby reshaping nasal immune homeostasis (**Figure 1**).

In the context of immune modulation, PRP may act through three primary pathways: (1) regulating mast cell activation and repair via factors like TGF-β1, (2) balancing the Th2/Th17/Treg axis to correct immune dysregulation, and (3) modulating neuroimmune interactions through components such as nerve growth factor (NGF) and (IL)-1 receptor antagonist (IL-1Ra). For barrier repair, PRP is hypothesized to promote structural regeneration (e.g., via PDGF, EGF, VEGF), mitigate damage through anti-inflammatory and antioxidant effects, and optimize the microenvironment via microcirculation improvement and matrix remodeling.

## 3 Evolution of the hypothesis: theoretical rationale

AR is a chronic nasal mucosal inflammatory disorder driven by allergens, with its core pathophysiological mechanisms involving multidimensional imbalances: IgE cross-linking of FcϵRI receptors on mast cells (MCs) triggers degranulation, releasing mediators (e.g., histamine, leukotrienes) and inducing acute symptoms (Deng et al., 8031); allergens activate Th0 cell differentiation into Th2 cells via dendritic cells (DCs), which secrete pro-inflammatory factors (e.g., IL-4, IL-13) to drive B-cell IgE production (Kang et al., 2023); Th17 cells mediate neutrophil infiltration, exacerbating chronic inflammation (Yu Mm and Yan Mm, 2023); and loss of tight junction proteins combined with goblet cell hyperplasia in the nasal mucosal epithelium disrupts barrier function, promoting allergen penetration and sustained inflammation (Yang et al., 2023; Li et al., 2025). Based on the established pathophysiology of AR, we hypothesize that PRP may intervene in disease progression through two synergistic pathways—immune modulation and barrier repair—to restore nasal immune homeostasis. To clarify this hypothesis, we operationally define “immune modulation” as PRP’s potential to exert effects beyond broad anti‐inflammation by targeting specific cellular networks in AR, including the bidirectional regulation of mast cells, correction of Th2/Th17/Treg imbalance, and modulation of neuro‐immune interactions. While PRP’s general anti‐inflammatory properties are documented in wound healing and orthopedics (Shi et al., 2021; Gruber, 2025), its specific immunomodulatory role in AR remains a novel proposal. Similarly, “barrier repair” is defined not merely as theoretical tight‐junction restoration but as a functional, multidimensional process involving structural regeneration of the epithelium, mitigation of ongoing damage via anti‐inflammatory and antioxidant actions, and optimization of the repair microenvironment through enhanced microcirculation and extracellular matrix remodeling. Although PRP’s pro‐regenerative effects have been observed in atrophic rhinitis and skin healing models, its efficacy in functionally restoring the AR mucosal barrier awaits validation. Central to our hypothesis is the concept of “dual repair, “ which posits that the synergistic action of these two mechanisms could disrupt the vicious cycle of immune dysfunction → barrier damage → amplified immune response in AR, thereby offering a comprehensive therapeutic strategy beyond single‐target approaches.

### 3.1 Proposed immune modulation: targeting multicellular interaction networks to restore immune homeostasis

PRP may exert immune modulation primarily by regulating mast cell function, the Th2/Th17/Treg axis, and neuroimmune microenvironments (Hemmer et al., 2024)—core to blocking the “allergen-IgE-MCs-Th2 inflammation” cycle and restoring immune tolerance.

#### 3.1.1 Targeting MCs: bidirectional regulation of activation/repair (modulating AR symptoms)

MCs are central effectors of acute AR inflammation; their aberrant activation (e.g., by IgE/IL-33 co-stimulation) releases histamine, TNF-α, and IL-6, directly inducing pruritus and sneezing (Deng et al., 8031). Based on evidence from wound healing and orthopedics (Shi et al., 2021; Gruber, 2025), it is postulated that TGF-β1 in PRP plays a critical bidirectional role in mast cell regulation. It suppresses MCs activation and pro-inflammatory cytokine release (Bao et al., 2021). Ndaw et al (Ndaw et al., 2017). demonstrated that TGF-β1 concentration-dependently blocked the release of TNF-α, IL-6, IL-13, and MCP-1 from IL-33/IgE-stimulated mast cells by inhibiting Akt/ERK phosphorylation and NF-κB/AP-1 transcription. In allergic rhinitis, IL-33 from epithelial cells and allergen-induced IgE act synergistically to activate mast cells, a process disrupted by TGF-β1, thereby reducing acute mucosal inflammation such as edema and pruritus. Ganeshan et al (Ganeshan and Bryce, 2012). reported that TGF-β1 inhibits IgE-dependent degranulation and histamine release via cAMP signaling; it also modulates mast cell IL-6 secretion to control neutrophilic inflammation, which may contribute to disease resolution (Kim and Lee, 1999; Ganeshan and Bryce, 2012). Conversely, TGF-β1 may exacerbate AR by activating MCs. Nakano et al (Nakano et al., 2021). found TGF-β/Notch signaling promotes mucosal MCs maturation—mucosal MCs, the dominant nasal MCs subtype, are hyperresponsive to allergens (e.g., IgE) and release more histamine/tryptase (mMCP-1). Elevated TGF-β1 in AR nasal mucosa may prime mucosal MCs for robust degranulation, intensifying pruritus/sneezing. Meurer et al (Meurer et al., 2025). noted TGF-β1 upregulates Mcpt1/Mcpt2 (tryptase/chymase) transcription; tryptase not only marks activation but also exacerbates AR via inflammation and vascular permeability. Zhao et al (Zhao et al., 2020). revealed a Treg-MCs positive feedback loop: MCs-secreted TGF-β1 expands Tregs, while Tregs maintain MCs survival via IL-9. Excessive TGF-β1 may disrupt this balance—suppressing degranulation but enhancing survival—leaving MCs “viable but active, “ continuously releasing low-level IL-4 to sustain Th2 inflammation and delay remission.

Additionally, the direction of TGF-β1’s effect—whether it suppresses or activates mast cells—likely depends on contextual triggers within the AR microenvironment, such as the presence of specific cytokines [e.g., IL-33, which may synergize with TGF-β1 to enhance activation via Notch signaling (Nakano et al., 2021)] or the balance of Treg-derived factors [e.g., IL-9 (Zhao et al., 2020)]. For instance, in an IL-33-rich milieu, TGF-β1 might favor mast cell inhibition, whereas in conditions of Notch pathway upregulation, it could promote maturation and hyperresponsiveness. These factors highlight the complexity of TGF-β1’s bidirectional role and underscore the need to evaluate PRP’s effects under varying cytokine conditions in AR models.

In summary, the hypothesis proposes that PRP modulates MCs activation through multiple targets, offering broader regulatory potential than single-pathway inhibitors. However, this bidirectional effect remains to be specifically validated in AR models.

#### 3.1.2 Regulating the Th2/Th17/treg axis and modulating neuroimmunity to correct imbalance

AR stems from immune dysregulation: Th2/Th17 overactivation and Treg hypofunction (Huber et al., 2021; Haque et al., 2024). PRP is rich in cytokines, including TGF-β1, which is known to promote Treg differentiation. Based on this established biology, we hypothesize that PRP may function as an immune modulator in AR. Tregs suppress Th2/B cells via IL-10/TGF-β1; AR patients exhibit elevated Th2/Th17/Treg percentages, peripheral eosinophils/basophils, and serum IL-4/IL-5/IL-17/IL-10/IgE vs. healthy controls (Guo et al., 2024)—suggesting PRP restores balance by replenishing Tregs and modulating Th1/Th2/Th17/Treg axes (Song et al., 2020). Additionally, platelet activation drives allergic inflammation; PRP may regulate immunity by releasing platelet-derived IL-33 (Takeda et al., 2016; Chen et al., 2017). Oxidative stress exacerbates Th1/Th2 and Treg/Th17 dysregulation (Cui et al., 2019); PRP’s antioxidants may indirectly restore balance by inhibiting oxidative stress (Yang et al., 2025; Yang et al., 2026); PRP also contains neurotrophic factors (e.g., NGF) that promote neurogenesis/angiogenesis with PDGF/VEGF (Minnone et al., 2017; Valente et al., 2022; Ali et al., 2025). While NGF’s role in trigeminal nerve substance P release is unreported, its modulation of neuronal activity/neuropeptide release supports efficacy against neurogenic symptoms (pruritus, sneezing). The leukocyte content significantly alters PRP’s cytokine profile. While leukocyte-rich PRP (L-PRP) expresses higher levels of the anti-inflammatory interleukin-1 receptor antagonist (IL-1Ra) compared to leukocyte-poor PRP (P-PRP) (Jayaram et al., 2023), this potential anti-inflammatory advantage must be weighed against the risk that L-PRP’s high neutrophil load could exacerbate AR inflammation. Therefore, the net effect in AR is unpredictable and underscores the need for subtype-specific investigation. IL-1Ra suppresses neuroimmune hyperexcitation upstream by reducing glia-mediated neuropeptide synthesis (Redondo-Castro et al., 2017; Jayaram et al., 2023). Together, NGF (direct neural modulation) and IL-1Ra (indirect glial inhibition) synergistically alleviate neurogenic symptoms.

### 3.2 Proposed barrier repair: multidimensional reconstruction of nasal mucosal epithelial integrity

Nasal mucosal barrier dysfunction is a key driver of AR chronicity and recurrence. PRP could reconstruct barrier repair via synergistic growth factor networks, targeting cellular repair, inflammation control, and microenvironment optimization.

#### 3.2.1 Activating epithelial repair signaling to promote structural regeneration

Drawing from its efficacy in atrophic rhinitis (Friji et al., 2014; Mostafa and Ayad, 2020; Kim et al., 2021; Bondarenko and Bezshapochny, 2023; Asiry et al., 2025; Tulaci et al., 2025), we propose that the mechanism by PRP might promote regeneration of nasal mucosa involves multi-pathway and multi-factor synergistic interactions. Its core lies in the high concentration of growth factors and cytokines in PRP, which regulate cellular behavior and the tissue microenvironment through paracrine and autocrine signaling (Wu et al., 2025). The synergistic effects of these factors activate complex signaling networks, promoting collagen synthesis, cell migration, and angiogenesis (Wu et al., 2025). They directly stimulate the proliferation and differentiation of nasal mucosal epithelial cells, fibroblasts, and vascular endothelial cells, effectively reversing mucosal atrophy, promoting ciliary regeneration, and restoring goblet cell function, thereby improving mucus properties and secretion volume (Friji et al., 2014; Mostafa and Ayad, 2020; Kim et al., 2021; Bondarenko and Bezshapochny, 2023; Asiry et al., 2025; Tulaci et al., 2025).

#### 3.2.2 Suppressing inflammation and oxidative stress to mitigate barrier damage

Studies in other inflammatory models suggest that PRP may mitigate barrier injury by potentially inhibiting TLR4/NF-κB signaling (Kang et al., 2023). Its antioxidant components, including superoxide dismutase, lower reactive oxygen species and malondialdehyde levels to alleviate oxidative damage (Tognoloni et al., 2023). Synergistic factors—PDGF for fibroblast proliferation, VEGF for angiogenesis, and IL-6 for inflammation regulation—act in concert with TGF-β1. This interaction balances pro-repair processes, such as PDGF/TGF-β1-mediated matrix repair and VEGF-driven perfusion, with the anti-inflammatory effects of IL-6 (Ganeshan and Bryce, 2012).

#### 3.2.3 Improving microcirculation and matrix remodeling to optimize repair

Based on its actions in other tissues, PRP is hypothesized to enhance local microcirculation by upregulating nitric oxide synthase (NOS) and arginase activity. This could promote nitric oxide (NO) production, thereby inducing vasodilation and angiogenesis, and improving tissue perfusion and oxygenation to supply essential nutrients for mucosal repair (Asiry et al., 2025). In extracellular matrix remodeling, PRP stimulates collagen synthesis, thereby reinforcing tissue strength and elasticity. Furthermore, growth factors in PRP, such as epidermal growth factor (EGF) and insulin-like growth factor-1 (IGF-1), may directly contribute to barrier repair by promoting tight junction protein recovery and epithelial proliferation. For instance, EGF has been shown to protect intestinal barrier integrity by binding to the EGF receptor and activating pathways like Ras/MAPK/ERK, which reduces the negative effects of oxidative stress on intercellular junctions (Tang et al., 2016). This mechanism could translate to the nasal mucosa, where EGF might enhance the expression of tight junction proteins (e.g., occludin and claudin-1) under inflammatory conditions, thereby restoring epithelial integrity. Similarly, IGF-1 exhibits dose-dependent mitogenic effects on olfactory epithelium; in aged mice, low-dose IGF-1 administration increased the numbers of olfactory progenitors and mature neurons, suggesting its potential to stimulate nasal epithelial cell proliferation and differentiation in AR (Ueha et al., 2018). These effects align with PRP’s proposed role in activating repair signaling through multi-factor synergy. Clinical studies have also observed a marked decrease in nasal hydrogen sulfide (H_2_S)—a toxic byproduct—following PRP treatment (Bondarenko and Bezshapochny, 2023), a reduction associated with improved mucosal integrity, suppressed microbial metabolism, and enhanced endogenous detoxification capacity. Collectively, these mechanisms underscore PRP’s capacity to optimize the repair microenvironment through coordinated actions on microcirculation, matrix remodeling, and growth factor-driven epithelial regeneration.

In summary, PRP targets allergic rhinitis through two synergistic pathways: immune modulation, by suppressing excessive mucosal MCs activation, correcting Th2/Th17/Treg imbalance, and regulating neuroimmune interactions; and barrier repair, through epithelial regeneration, inflammation inhibition, and microenvironment optimization via coordinated growth factor actions. It is crucial to note that the theoretical rationale outlined below extensively incorporates mechanistic insights from *in vitro* studies, research on non-nasal tissues (e.g., dermal, orthopedic), and inflammatory conditions distinct from AR. While these data provide a compelling biological plausibility for our hypothesis, they serve as a supportive rationale rather than direct evidence for PRP’s efficacy in AR. The applicability of these mechanisms within the specific immunological and anatomical context of the nasal mucosa in AR remains a central premise of this hypothesis and requires dedicated experimental validation.

## 4 Hypothesis validation pathway

To test the proposed hypothesis, a stepwise investigative approach will be adopted, following a “mechanism dissection → model evaluation → clinical translation” logic. Gradient experiments, centered on the dual pathways of “immune modulation” and “barrier repair, “ will be conducted to systematically explore PRP’s potential intervention in AR pathogenesis (**Table 1**).

**Table 1:** Gradient experimental design to validate PRP’s intervention in AR pathogenesis via dual pathways (“immune modulation” and “barrier repair”).

Validation level	Experiment type	Model/subject	Core treatment	Key detection indicators	Validation goals
*In Vitro* Experiments	Mast Cell Co-Culture	Human HMC-1 mast cells	PRP treatment at 1%, 5%, 10% concentrations	FcϵRI expression, calcium influx, histamine/IL-6 release; c-Kit/Foxp3 expression (differentiation/repair markers)	Elucidate PRP’s bidirectional effects: inhibition of mast cell activation and promotion of repair.
	T Cell Differentiation Assay	Naïve CD4^+^ T cells	PRP-conditioned medium in serum-free, cytokine-free medium	Th2/Th17/Treg cell ratios; cytokine secretion (IL-4/IL-17/IL-10); Foxp3 expression	Investigate PRP’s regulation of Th2/Th17/Treg axis imbalance.
	Neuroimmune/Oxidative Stress	Human HAPI glial cells Human nasal epithelial cells (HNECs)	PRP treatment on HAPI; H_2_O_2_-induced oxidative stress + PRP	Neuropeptide synthesis (SP, CGRP, POMC); GFAP expression (glial activation); ROS/SOD/MDA levels	Clarify IL-1Ra’s inhibition of neuroimmune hyperactivity and PRP’s antioxidative capacity.
	Epithelial Barrier Repair	RPMI 2650 nasal epithelial cells	PRP treatment on scratch-wounded monolayers	Occludin/Claudin-1 expression; wound closure rate	Validate PRP’s capacity to enhance epithelial integrity and barrier function.
Animal Experiments	OVA-Induced AR Mouse Model	OVA-sensitized AR mouse model	Control (PBS); PRP low-dose/high-dose	Behavioral endpoints (sneezing/scratching); serum OVA-IgE/Th2/Th17/Treg cytokines; mucosal histology (H&E, immunofluorescence for Occludin/Foxp3)	Evaluate PRP’s systemic effects on immune dysregulation and barrier damage in AR.
Clinical Pilot	Safety/Efficacy Evaluation	Refractory AR patients	Unilateral intranasal PRP injection	Adverse events (local/systemic); RQLQ/TNSS/VAS scores; PNIF; mucosal cytokines (IL-4/IL-13) and Foxp3/Occludin expression	Assess PRP’s preliminary safety, symptom relief, and molecular efficacy in humans.

### 4.1 *In vitro* experiments: exploring PRP’s functional effects on immune and epithelial cells

PRP will be prepared from healthy adult donors via peripheral blood collection. Using differential centrifugation (900 × *g* for 5 min, followed by 1, 500 × *g* for 15 min), platelet-rich plasma will be isolated and aliquoted aseptically (Tian et al., 2019). Platelets will be maintained in a non-activated state to preserve native growth factor profiles, with concentrations standardized to ≥1 × 10^6^ platelets/μL for consistency. Finally, PRP will be activated with thrombin and calcium gluconate (Tian et al., 2019).

Based on the theoretical rationale of the hypothesis, a series of *in vitro* assays are proposed to mechanistically investigate PRP’s potential effects on immune and epithelial cells. Human mast cells (HMC-1) will be co-cultured with PRP at varying concentrations (1%, 5%, 10%) in serum-free, cytokine-free medium (to minimize confounding variables) to assess its influence on FcϵRI expression, calcium influx, and the release of histamine and IL-6—metrics indicative of mast cell activation inhibition (Deng et al., 8031). Furthermore, the expression of differentiation markers (e.g., c-Kit, FcϵRI) and Treg-associated factors (FOXP3) will be quantified to examine the postulated bidirectional role of TGF-β1 in mast cell regulation and repair (Zhao et al., 2020; Jiao et al., 2021).

A separate assay will focus on CD4^+^ T cell differentiation to explore PRP’s immunomodulatory potential on T-cell polarization, explicitly distinct from the HMC-1 and PRP co-culture described above. Naive CD4^+^ T cells will be cultured in PRP-conditioned medium, with flow cytometry used to determine Th2, Th17, and Treg cell proportions and Enzyme-Linked Immunosorbent Assay (ELISA) to measure secreted IL-4, IL-17, and IL-10 levels, thereby testing the hypothesis that PRP can modulate the Th2/Th17/Treg balance (Tran et al., 2020).

In neuroimmune and oxidative stress models, human astrocytes (HAPI) will be treated with PRP to evaluate its impact on neuropeptide synthesis (e.g., proopiomelanocortin [POMC], calcitonin gene-related peptide [CGRP] precursor, substance P [SP]) and glial cell activation (e.g., glial fibrillary acidic protein [GFAP]), probing the proposed inhibitory role of IL-1Ra (Jayaram et al., 2023). For oxidative stress assessment, nasal mucosal epithelial cells will first be subjected to H_2_O_2_-induced injury (to mimic oxidative damage) and then treated with PRP—aligning with clinical use of PRP as a post-injury therapy rather than preventive agent. Changes in Reactive Oxygen Species (ROS), superoxide dismutase (SOD), and malondialdehyde (MDA) levels will be measured to assess PRP’s potential antioxidant capacity (Tognoloni et al., 2023).

Finally, in an epithelial barrier repair model, a scratch wound assay using human nasal epithelial cells (e.g., RPMI 2650 (Wong et al., 2024)) will be established with PRP addition to assess the rate of epithelial monolayer wound closure and structural regeneration. Complementary Transwell assays will be performed in parallel to quantitatively evaluate the functional restoration of the nasal epithelial barrier: PRP-treated and untreated nasal epithelial cells will be seeded onto the upper chambers of Transwell inserts with porous membranes, and the transepithelial electrical resistance (TEER) will be measured at multiple time points to assess barrier integrity, while the paracellular permeability of a fluorescent tracer across the cell monolayer will be detected by measuring fluorescence intensity in the lower chambers. Additionally, Western blot analysis of the tight junction proteins Occludin and Claudin-1 will be conducted for both experimental setups to determine the molecular mechanisms underlying PRP-mediated barrier repair, collectively validating PRP’s capacity to promote both the structural regeneration and functional restoration of the nasal epithelial barrier.

### 4.2 Animal experiments: evaluating PRP’s effects in an AR model

To verify the *in vivo* validity of the hypothesis, an ovalbumin (OVA)-induced AR model will be established using 6–8 weeks old female BALB/c mice (18–22 g, SPF grade). Mice will be sensitized by intraperitoneal injection of OVA-aluminum hydroxide emulsion on days 0, 7 and 14, followed by intranasal OVA challenge from days 21 to 27; normal control mice will receive equal-volume Phosphate-Buffered Saline (PBS) for both sensitization and challenge (Sun et al., 2022).

Mice will be randomly divided into three groups (n=18 per group): PBS control group (intranasal PBS instillation during OVA challenge), low-dose PRP group (5% autologous PRP) and high-dose PRP group (10% autologous PRP). All mice will receive daily intranasal instillation for 7 consecutive days under light isoflurane anesthesia. Autologous PRP will be prepared from naive BALB/c mice via two-step centrifugation, and its platelet concentration will be verified to be 3–5 times that of whole blood prior to use.

A 7-day observation period will be conducted during the intervention. Sneezing and nasal scratching frequency will be recorded daily within 30 min after challenge to assess AR behavioral symptoms. On day 28 (24 h after the last intervention), mice will be euthanized for sample collection: orbital sinus blood will be collected for serum separation (to detect OVA-specific IgE and cytokines IL-4/IL-13/IL-17/IL-10 by ELISA); nasal turbinate tissues will be dissected for H&E staining (to evaluate inflammatory cell infiltration), immunofluorescence (to examine Occludin/Foxp3 expression) and flow cytometry (to quantify the nasal mucosal Th2/Treg ratio). These assays are designed to determine whether PRP can ameliorate immune dysregulation and nasal epithelial barrier damage in AR mice, and to verify the *in vivo* effect of PRP-mediated “immune-barrier dual repair”.

### 4.3 Clinical study: a pilot investigation of safety and preliminary effects

A prospective, single-center, randomized, double-blind, placebo-controlled pilot study can be conducted. The study will enroll a sufficient number of adult participants (targeting a sample size of >30 subjects) with a confirmed diagnosis of moderate-to-severe, persistent refractory allergic rhinitis AR who have exhibited an inadequate response to standard intranasal corticosteroid therapy.

Participants will be randomly assigned to receive either unilateral intranasal injection of autologous PRP in the intervention group or sterile saline (placebo) in the control group. All procedures will be performed under endoscopic guidance to ensure precision and safety. The study design includes a baseline assessment period followed by a post-intervention follow-up extending over several weeks. Safety monitoring will focus on local reactions (erythema, edema, pain) and systemic adverse events, assessed at multiple time points. Safety monitoring will focus on local reactions (erythema, edema, pain) and systemic adverse events, assessed at multiple time points. Efficacy evaluations will incorporate patient-reported outcomes—including the Rhinoconjunctivitis Quality of Life Questionnaire (RQLQ), Total Nasal Symptom Score (TNSS), and Visual Analogue Scale (VS)—as well as objective physiological parameters such as Peak Nasal Inspiratory Flow (PNIF) and nasal airflow measurements (Li et al., 2021). To investigate the proposed mechanisms of action, nasal mucosal biopsies will be collected from consenting participants at baseline and during follow-up. Specifically, expression of tight junction proteins (e.g., Occludin) will be analyzed from these mucosal tissue specimens. Additional analyses will assess changes in Th2 cytokines (IL-4, IL-13) and Treg markers (Foxp3) to evaluate the immunomodulatory and barrier-repair effects of PRP.

In summary, this stepwise approach—*in vitro* mechanistic exploration, *in vivo* model evaluation, and pilot clinical investigation—is intended to systematically test the hypothesis of PRP’s dual-pathway activity in AR. The data generated from these exploratory studies would help inform the design of future multi-center, large-sample randomized controlled trials, which would be necessary to rigorously assess long-term efficacy, optimal dosing, and safety.

## 5 Discussion

Current first-line management of AR remains symptom-control oriented: intranasal corticosteroids (INCS) alleviate inflammation by suppressing Th2 cytokine secretion, but long-term use risks nasal mucosal atrophy and local immune suppression (Chitsuthipakorn et al., 2022); second-generation antihistamines relieve symptoms via histamine H_1_ receptor antagonism but fail to repair the nasal mucosal physical barrier; allergen immunotherapy (AIT) requires ≥3 years of continuous treatment to induce long-term immune tolerance (Shamji et al., 2022); and emerging biologics, while targeting specific immune pathways, have critical limitations—they address only single molecular targets (unable to correct multidimensional Th2/Th17/Treg imbalance) and lack tissue regenerative capacity (failing to reverse structural nasal mucosal damage). None of these therapies simultaneously target the core AR pathology: the vicious cycle of “immune imbalance-barrier damage.” This leads to high post-treatment recurrence rates, creating an urgent need for strategies that combine “immune modulation + tissue regeneration.” (Yamani et al., 2024).

The core innovation of this hypothesis is proposing PRP as a therapy that breaks through traditional treatment bottlenecks via an “immune-barrier dual repair” mechanism: synergistic action of its multiple factors can simultaneously correct immune dysregulation (inhibit Th2/Th17 cells, promote regulatory T cells [Tregs]) and restore the physical barrier (enhance epithelial regeneration). This may disrupt the “inflammation-barrier damage” cycle and reduce recurrence. Critically, PRP’s autologous origin avoids the immune rejection risks of allogeneic biologics, offering superior safety to current targeted therapies. Furthermore, prior studies confirm PRP promotes olfactory bulb regeneration in patients with olfactory dysfunction (Kim et al., 2025), suggesting it could improve AR-associated olfactory loss by repairing olfactory mucosa. If validated, PRP could offer a novel therapeutic alternative for three key patient groups: those with mild-to-moderate AR; severe AR patients with comorbid nasal polyps or airway hyperresponsiveness; and children or pregnant women who are hesitant about the safety of long-term pharmacotherapy, an understandable concern given that autologous PRP eliminates the risk of drug residues. Furthermore, a key limitation underlying this hypothesis is the reliance on extrapolation from non-AR models. The mechanistic links proposed, while biologically plausible, are primarily supported by evidence from *in vitro* systems, wound healing, orthopedics, and other inflammatory conditions. The direct relevance and relative contribution of these pathways in the complex pathophysiology of AR necessitate rigorous validation in AR-specific models, as outlined in our validation pathway.

However, the clinical translation of PRP for AR still faces significant challenges, which must be thoroughly considered. A primary concern is PRP heterogeneity; its biological effects are not uniform but are critically dependent on preparation protocols (Al-Hamed et al., 2021; Wu et al., 2021; Acebes-Huerta et al., 2024). Specifically, the leukocyte content—distinguishing L-PRP from P-PRP—demands careful consideration. Given that AR is characterized by eosinophilic and Th2-driven inflammation, the high neutrophil load in L-PRP could potentially exacerbate the inflammatory milieu. Therefore, this hypothesis specifically focuses on P-PRP, which is theorized to offer a more favorable safety profile by minimizing the risk of amplifying neutrophilic inflammation, though this remains to be validated. Additional challenges include: issues with bioactivity stability, as the short half-life of active factors released from platelet α-granules (Khandan-Nasab et al., 2025) necessitates optimized preservation techniques; the difficulty of standardization due to influence from individual biological characteristics (age, sex) and preparation parameters (centrifugation speed, anticoagulant type) (Xiong et al., 2018; Mochizuki et al., 2023; Pineda-Cortel et al., 2023); and limitations in administration routes, highlighting the need for future development of sustained-release formulations. Regarding clinical positioning and safety, the speculative applicability of PRP across diverse patient populations, such as children or pregnant women, is noted here as a theoretical consideration based on its autologous nature, but it is acknowledged that this positioning is premature in the absence of AR-specific clinical data. A paramount safety consideration involves patients with AR and comorbid asthma, which represents a distinct disease phenotype (Bousquet et al., 2023). The systemic immunomodulatory effects hypothesized for PRP could potentially lead to unpredictable fluctuations in pulmonary inflammation in these patients. Consequently, extreme caution is warranted, and initial clinical investigations should prioritize otherwise healthy adults with AR to first establish a foundational safety profile.

In summary, this hypothesis proposes a novel “immunomodulation + tissue regeneration” paradigm for AR treatment, facilitating the transition of PRP from empirical application to mechanism-driven therapy. Future research should focus on three key directions: spatiotemporal regulation of critical active components—utilizing single-cell sequencing to elucidate the dynamic regulatory networks of TGF-β1 and EGF in PRP on Th17/Treg polarization; adaptation to the nasal mucosal microenvironment—establishing 3D biomimetic nasal mucosal chips to simulate PRP efficacy under varying pH (6.5–7.8) and inflammatory cytokine (IL-4/IL-13) gradients; and optimization of combination therapy—exploring the integration of PRP with other treatment modalities.

## 6 Conclusion

PRP employs a dual mechanism for immune barrier repair that targets the Th2/Th17/Treg axis imbalance, representing an innovative approach to AR treatment. Current *in vitro* and animal studies support its immunomodulatory capacity and ability to restore the nasal mucosal barrier, yet high-quality clinical trials are necessary to confirm its efficacy and safety. This hypothesis offers a fresh perspective on AR pathogenesis and establishes a theoretical basis for developing novel therapies that address both the symptoms and root causes of AR.
